# Differential expression profile of urinary exosomal microRNAs in patients with mesangial proliferative glomerulonephritis

**DOI:** 10.18632/aging.204527

**Published:** 2023-02-14

**Authors:** Rong Dai, Lei Zhang, Hua Jin, Dong Wang, Meng Cheng, Yunhui Xu, Haiyin Zhang, Yiping Wang

**Affiliations:** 1 Department of Chinese Medicine, Anhui University of Chinese Medicine, Hefei 230038, China; 2 Department of Nephrology, The First Affiliated Hospital of Anhui University of Chinese Medicine, Hefei 230031, China; 3 Graduate School, Anhui University of Chinese Medicine, Hefei 230038, China

**Keywords:** epigenetic, exosome, microRNA, mesangial proliferative glomerulonephritis

## Abstract

Objective: To investigate the differential expression profile of urinary exosomal microRNA (miRNA) in patients with mesangial proliferative glomerulonephritis (MsPGN) and healthy controls and their potential role in the pathogenesis of MsPGN.

Methods: Urine specimens were collected from five MsPGN patients and five healthy controls, and differentially expressed miRNAs were screened using high-throughput sequencing technology. The sequenced urinary exosomal miRNAs were further investigated by quantitative real-time polymerase chain reaction (qRT-PCR) in a validation cohort (16 MsPGN patients and 16 healthy controls). Correlation and receiver operating characteristic (ROC) curve analyses were used to determine the association between clinical features and miRNA expression in MsPGN. Finally, fluorescence *in situ* hybridization was performed to detect miRNA expression in the renal tissues of MsPGN patients.

Results: Five differentially expressed miRNAs (miR-125b-2-3p, miR-205-5p, let-7b-3p, miR-1262, and miR-548o-3p) were identified by qRT-PCR. The expression of these miRNAs correlated with ACR, 24hUpro, mAlb, UA, and combined yielded a ROC curve area of 0.916 in discriminating MsPGN patients from the controls. In addition, the expression of miR-205-5p, let-7b-3p, miR-1262, and miR-548o-3p was elevated in the MsPGN patient group, and miR-125b-2-3p was decreased in the MsPGN patient group.

Conclusions: Differential expression of urinary exosomal miRNAs may pose a risk of MsPGN and help distinguish MsPGN patients from controls. Certain miRNA expressions may be associated with disease progression, contributing to the epigenetic understanding of the pathophysiology of MsPGN.

## INTRODUCTION

Mesangial proliferative glomerulonephritis (MsPGN), one of the most important kidney diseases, is characterized by apoptosis, proliferation, and extracellular matrix (ECM) secretion of glomerular mesangial cells (GMCs) and is representative of nephritis leading to chronic kidney disease (CKD) [1, 2]. Furthermore, MsPGN accounts for approximately 10.5% of primary glomerulonephritis diseases in China and can develop into glomerulosclerosis, interstitial fibrosis, and end-stage renal disease [3]. The etiology of MsPGN is unclear, and some studies have reported that it is a severe immune-mediated inflammatory disease caused by the deposition of immune circulating complexes in the glomerular thylakoid membrane through some antigens, which stimulate the body to produce antibodies [4]. To date, the pathogenesis of MsPGN has not been well elucidated.

Epigenetic mechanisms can affect gene expression and function without altering the underlying DNA sequence and mediate crosstalk between genes and the environment [5]. Unlike dichotomous genetic variation, they provide continuous regulation of the genetic effects of phenotypic changes. Epigenetic regulation includes non-coding RNAs, DNA hypo- or hypermethylation, histone modifications, and heterochromatin [6]. MicroRNAs (miRNAs) are single-stranded non-coding RNAs consisting of 18–25 nucleotides that can repress specific target messenger RNAs (mRNAs) by cleavage or translation and post-transcriptionally regulate gene expression variously, with significant biological functions in the physiology and pathology of various diseases [7]. Exosomes are extracellular vesicles with a diameter of 40–160 nm (average 100 nm), comprising lipids, proteins, and nucleic acids, including mRNA, miRNA, or long-stranded non-coding RNA [8]. They are present in almost all biological fluids, including urine [9].

Currently, renal biopsy is the gold standard for diagnosing MsPGN; however, since it is an invasive method, repeated renal biopsies are not significantly effective in assessing disease severity and progression. It has been shown that a few miRNAs are highly expressed in the plasma of MsPGN patients compared with that of healthy controls, highlighting the possible presence of altered miRNA levels in the disease that could serve as a potential novel diagnostic biomarker for MsPGN [10]. Urinary exosomes are composed of proteins, mRNA and miRNA produced by glomerular cells (podocytes, endothelial cells, and GMCs), and renal tubular cells [11]. Thus, urinary exosomes may provide sensitive and accurate biomarkers of renal dysfunction and structural damage [12]. Urine is a suitable sample source for isolating and extracting exosomes, as it is easily accessible.

Numerous studies have revealed different urinary exosome miRNA expression patterns in patients with kidney disease. Urinary exosomes miR-29c, miR-146a, and miR-205 may serve as IgAN biomarkers [13], while miR-3135b, miR-654-5p, and miR-146a-5p are candidate biomarkers for combined cellular crescent in type IV lupus nephritis [14], and miR-146a may be a potential indicator of early renal injury in hypertension [15]. However, the MsPGN urinary exosome miRNA expression profile is yet to be elucidated. Therefore, it is crucial to explore new noninvasive diagnostic biomarkers. MiRNA expression is differentially expressed in circulating exosomes in MsPGN, which could result in aberrant regulatory information being transmitted to the target organs and functional abnormalities of associated genes and pathways. Considering the underlying mechanisms of multisystem diseases, studying exosomal miRNAs may reveal new targets and aberrant epigenetic regulation contributing to the risk of MsPGN. Therefore, in this study, we determined the expression profile of exosomal miRNAs in the urine of MsPGN patients to partially reveal the pathophysiology of the disease, which could be used to design prospective molecular targets for MsPGN diagnosis.

## RESULTS

### Clinical characteristics

The MsPGN patient and control groups participating in the sequencing and validation study and their clinical characteristics are summarized in Table 1. No statistically significant differences were observed between the MsPGN patient and control groups in age, sex, blood pressure, Alanine transaminase (ALT), Aspartate transaminase (AST), Blood urea nitrogen (BUN), Serum creatinine (Scr), urinary β2-microglobulin (β2-MG), urine red blood cell (URBC), Immunoglobulin G (IgG), and complement C3 (C3). However, there were significant differences in Total Protein (TP), albumin (ALB), Urine Albumin to. Creatinine Ratio (ACR), Urine Total Protein to. Creatinine Ratio (TCR), 24 hours urine protein (24hUpro), microalbuminuria (mAlb), uric acid (UA), Immunoglobulin A (IgA), Immunoglobulin M (IgM), and cystatin C (CysC) between the MsPGN patient and control groups.

**Table 1:** Clinical characteristics of MsPGN patients and the controls.

**Parameters**	**MsPGN**	**Control**	**t/χ^2^**	***P***	**Difference and 95% confidence interval**
Age	38.57±15.36	36.14±3.39	0.708	0.483	2.49 (-4.51–9.36)
Sex [M/F, *n*(%)	9(42.86)/12(57.14)	10(47.62)/11(52.38)			
Systolic blood pressure (mmHg)	121.57±14.37	120.19±1.81	0.437	0.664	1.38 (-5.01–7.76)
Diastolic blood pressure (mmHg)	79.86±16.38	80.29±1.90	-0.119	0.906	-0.43 (-7.70–6.85)
ALT (U/L)	24.10±14.65	23.76±6.70	0.095	0.925	0.33 (-6.77–7.43)
AST (U/L)	22.33±10.49	17.76±3.13	1.914	0.063	4.57 (-0.25–9.39)
TP (g/dL)	55.52±12.03	67.46±6.08	4.061	0.001	-11.94 (-17.88–5.99)
ALB (g/dL)	32.41±7.71	40.14±3.05	4.273	0.001	-7.72 (-11.38–4.07)
BUN	6.21±2.15	5.29±1.37	1.659	0.105	0.92 (-0.21–2.05)
Scr	118.35±152.67	50.03±4.26	2.05	0.054	68.32 (0.96–135.68)
ACR (mg/mmol)	1,557.92±2,216.29	12.40±4.07	3.196	0.005	1,545.51 (568.05–2,522.98)
TCR (mg/mmol)	2.57±3.39	0.09±0.04	3.350	0.003	2.48 (0.98–3.98)
24hUpro (g/24 h)	2.73±3.25	0.09±0.03	3.717	0.001	2.64 (1.21–4.07)
mAlb (mg/L)	1,617.42±1,704.14	9.73±1.06	4.323	0.001	1,607.69 (856.10–2,359.27)
β2-MG (mg/L)	0.60±1.11	0.009±0.007	1.627	0.114	0.60 (0.11–1.09)
UA (μmol/L)	424.57±96.51	217.37±52.33	8.606	0.001	206.20 (157.77–254.61)
URBC	347.07±992.84	1.96±0.09	1.593	0.127	345.10 (-92.77–782.99)
IgA (g/L)	2.45±0.91	0.98±0.56	6.317	0.001	1.48 (1.00–1.95)
IgG (g/L)	7.41±2.77	7.02±0.86	0.612	0.544	0.38 (-0.89–1.67)
IgM (g/L)	0.99±0.34	0.80±0.08	2.530	0.019	0.19 (-0.04–0.35)
C3 (mg/dL)	1.13±0.23	1.19±0.03	1.308	0.198	-0.06 (-0.17–0.04)
CysC (mg/L)	1.09±0.43	0.71±0.32	3.205	0.003	0.37 (0.13–0.61)

### Identification of isolated exosomes in urine

**Figure 1:**
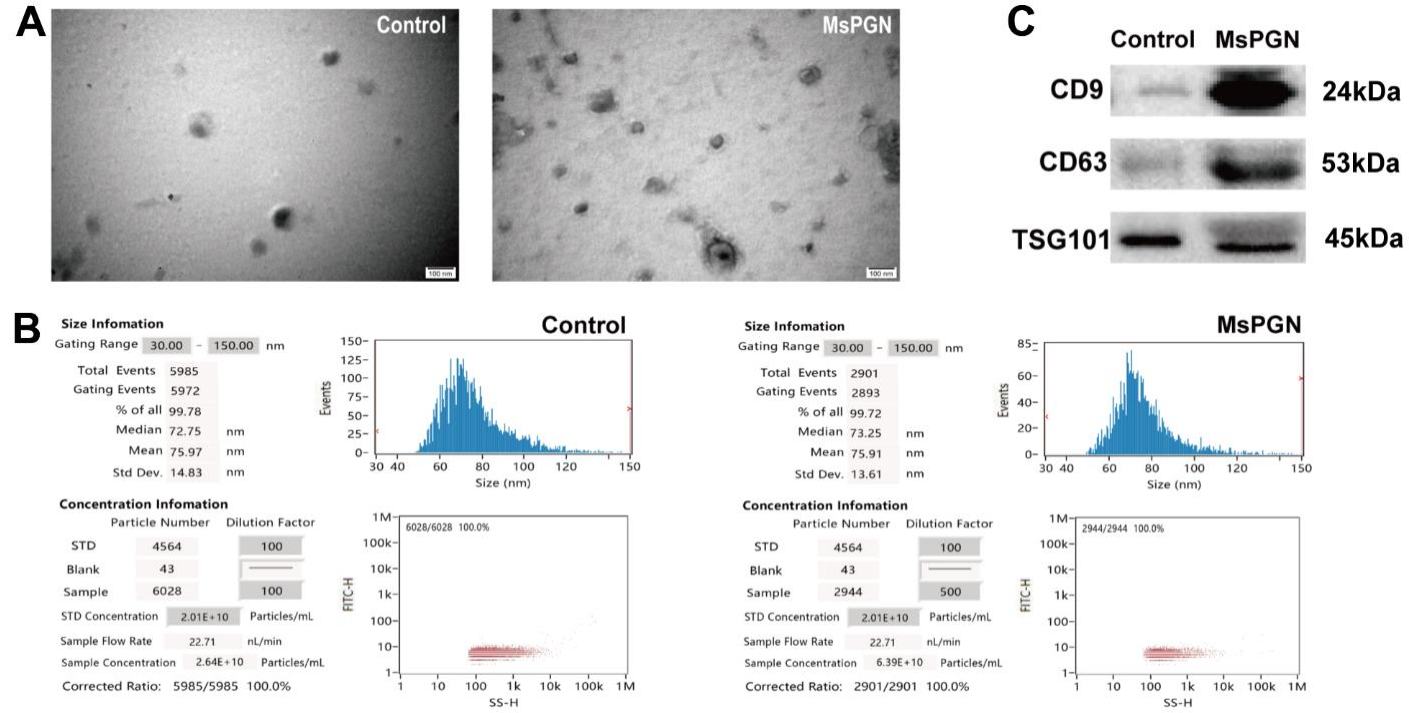
(**A**) Electron microscopic images of extracted exosomes revealed cup-shaped structures with a diameter of about 30–160 nm. Scale bar: 100 nm. (**B**) Nanoparticle tracking analysis (NTA) revealed the diameter of isolated extracellular vesicles (EVs) is consistent with that of exosomes. (**C**) Western blotting revealed CD9, CD63 and TSG101 proteins in exosome samples.

Exosomes from human urine are characterized by their morphology, diameter, concentration, and the presence of exosome-rich protein markers such as CD9, CD63, and TSG101. Transmission electron microscopy revealed exosomal bilayers of membranous structure and oval shape, with a typical cup-shaped structures, ranging in size from 30–100 nm. The results of particle size analysis of urinary exosomes in normal humans showed that the average particle size of urinary exosomes was 75.97 nm after 100-fold dilution, and a total of 5,985 particles were detected, with 99.78% of the particles being 30–150 nm and were at an average concentration of 2.64 × 10^10^ particles/mL. In patients with MsPGN, the mean particle size of urinary exosomes was 76.89 nm, and a total of 4,462 particles were detected, with 99.89% being 30–150-nm particles at a mean concentration of 3.93 × 10^11^ particles/mL. In addition, western blotting revealed that these exosomes were positive for CD9, CD63, and TSG101 proteins (Figure 1). CD9 and CD63 are exosome-enriched proteins of the tetraspanin family, whereas TSG101 is a protein essential for multivesicular body biogenesis.

**Figure 2:**
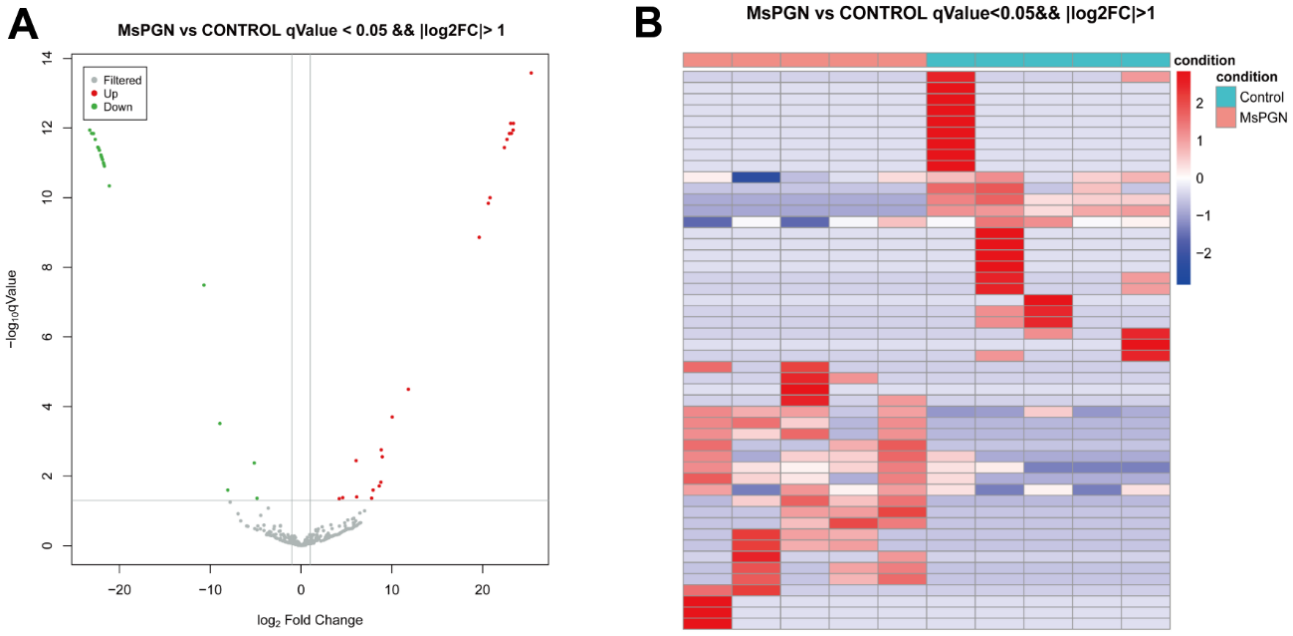
(**A**) volcano plot. (**B**) Heat map of 50 differentially expressed miRNAs (*P* < 0.05).

By comparing the TargetScan, miRDB, and miRWalk databases, 334, 445, and 279 miRNAs were found to be expressed in the normal control group, MsPGN patients, and co-expressed in both groups, respectively. The 50 significantly differentially expressed miRNAs (Table 2) were derived based on the log2 (fold change) absolute value ≥ 1, *q*-value < 0.05 screening criteria, among which 24 and 26 miRNAs were upregulated and downregulated, respectively (Figure 2A, 2B).

**Table 2:** Differentially expressed miRNAs in the normal control and MsPGN patient groups.

**miRNA_id**	**log2 fold change**	**q-value**	**Regulation**
hsa-let-7b-3p	10.00890681	0.000202	Up
hsa-let-7e-3p	8.577566075	0.019361167	Up
hsa-miR-1228-5p	-23.11264422	1.43E-12	Down
hsa-miR-125b-2-3p	6.027374621	0.003605977	Up
hsa-miR-1262	8.799320923	0.001767307	Up
hsa-miR-1273h-3p	23.34957173	7.40E-13	Up
hsa-miR-1293	-23.32349818	1.15E-12	Down
hsa-miR-1294	-22.08902223	5.97E-12	Down
hsa-miR-1343-3p	-21.91746382	8.12E-12	Down
hsa-miR-135a-5p	7.727456012	0.043054403	Up
hsa-miR-139-3p	-22.90038359	1.44E-12	Down
hsa-miR-181c-5p	-22.43428081	3.58E-12	Down
hsa-miR-181d-5p	4.555171863	0.041702492	Up
hsa-miR-193b-3p	8.908099501	0.002788228	Up
hsa-miR-205-5p	-4.897726616	0.043311736	Down
hsa-miR-211-3p	-22.95012868	1.44E-12	Down
hsa-miR-296-3p	23.32546696	1.15E-12	Up
hsa-miR-3126-3p	-22.25081379	4.37E-12	Down
hsa-miR-342-3p	7.900768613	0.025148193	Up
hsa-miR-3622a-5p	20.77214866	1.01E-10	Up
hsa-miR-3681-5p	-21.8178209	1.00E-11	Down
hsa-miR-371a-3p	-22.40844739	3.60E-12	Down
hsa-miR-374a-5p	6.095599185	0.039916143	Up
hsa-miR-378a-5p	-21.16264082	4.60E-11	Down
hsa-miR-3907	22.64562103	2.13E-12	Up
hsa-miR-3911	-21.76498145	1.11E-11	Down
hsa-miR-3936	19.58400166	1.39E-09	Up
hsa-miR-432-5p	20.60018108	1.47E-10	Up
hsa-miR-4665-5p	22.35871917	3.66E-12	Up
hsa-miR-4741	-22.09135782	5.97E-12	Down
hsa-miR-4745-5p	-22.95332405	1.44E-12	Down
hsa-miR-485-5p	-8.978971667	0.000308968	Down
hsa-miR-495-3p	-22.71936737	2.13E-12	Down
hsa-miR-5001-5p	-21.99068592	7.03E-12	Down
hsa-miR-5009-5p	25.30907609	2.63E-14	Up
hsa-miR-508-3p	4.156043977	0.044601029	Up
hsa-miR-509-3p	11.78208765	3.21E-05	Up
hsa-miR-548o-3p	8.758271119	0.014954382	Up
hsa-miR-576-3p	-22.34262498	3.81E-12	Down
hsa-miR-6510-3p	23.13492134	1.43E-12	Up
hsa-miR-6514-5p	-8.104544275	0.025148193	Down
hsa-miR-6721-5p	23.05569673	7.40E-13	Up
hsa-miR-6741-3p	22.90784403	1.44E-12	Up
hsa-miR-6770-3p	22.65026699	2.13E-12	Up
hsa-miR-6805-5p	-22.24849965	4.37E-12	Down
hsa-miR-6812-3p	-22.03235497	6.60E-12	Down
hsa-miR-7848-3p	-21.70172943	1.25E-11	Down
hsa-miR-891a-5p	-5.200989928	0.004204758	Down
hsa-miR-892a	-10.73226884	3.27E-08	Down
hsa-miR-942-5p	-22.96131874	1.44E-12	Down

### Validation of differentially expressed miRNAs

**Figure 3:**
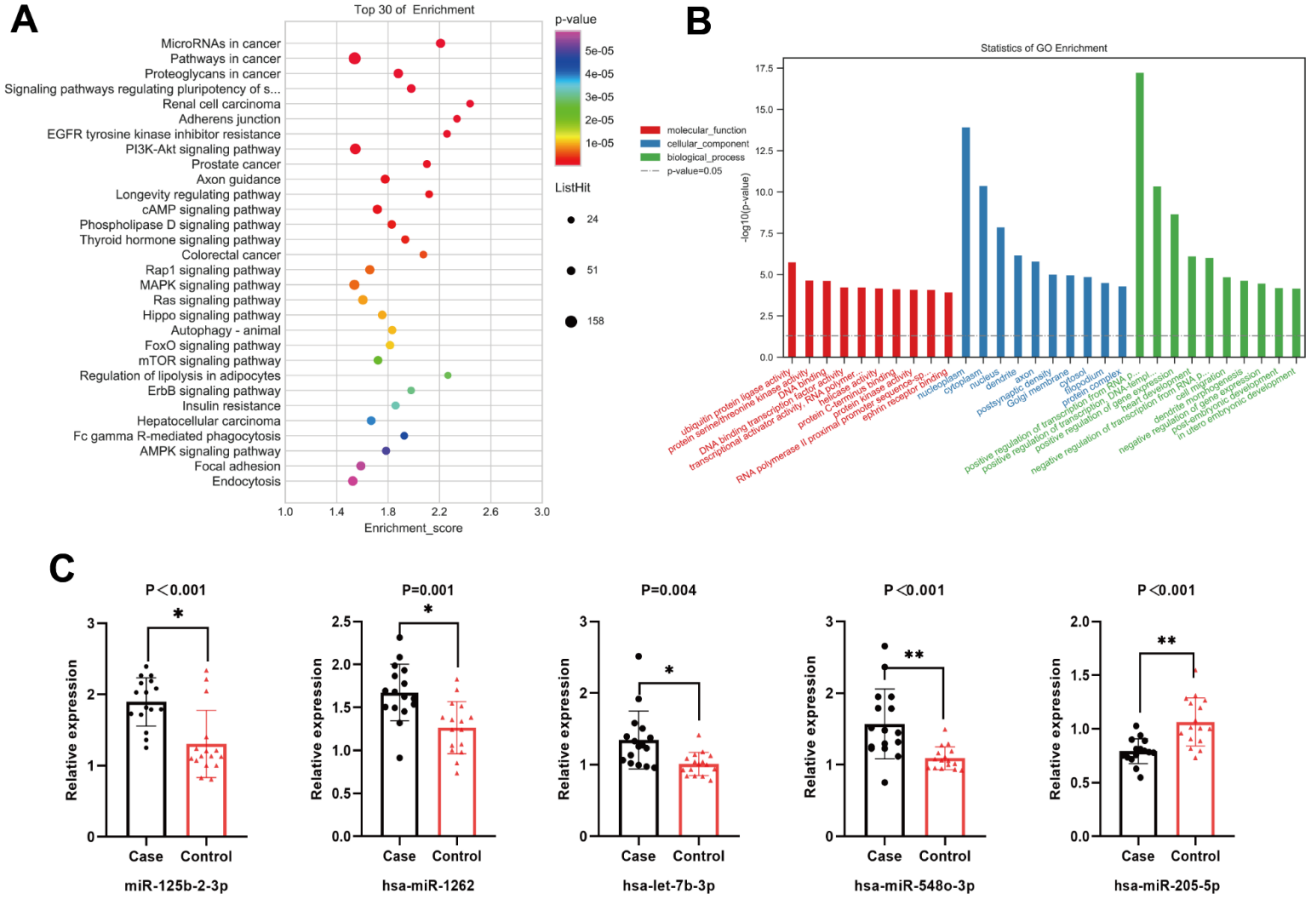
(**A**) Top 20 statistically significantly enriched pathways in Kyoto Encyclopedia of Genes and Genomes (KEGG) analysis. (**B**) Gene Ontology (GO) analysis for the target genes of five differentially expressed miRNAs. (**C**) The relative expression level of five differentially expressed miRNAs.

The validation cohort comprised a sample size of 32 (16 MsPGN patients and 16 healthy controls). To ensure the accuracy of the sequencing results, miRNAs for the subsequent PCR validation were screened according to the following criteria: (1) the mean expression (TPM) within at least one of the two groups of samples was > 2; (2) the absolute value of log2 (fold change) was ≥ 2; (3) at least one sample in the MsPGN group had a TPM > 0. The final selection of upregulated miR-125b -2-3p, miR-1262, miR-508-3p, let-7b-3p, miR-548o-3p, and miR-193b-3p and down-regulated miR-205-5p and miR-891a-5p were used for PCR validation. After statistical analysis, there were significant differences in the expression of miR-125b-2-3p, miR-1262, let-7b-3p, miR-548o-3p, miR-205-5p, and miR-193b-3p in the MsPGN group compared with that in the normal control group (*P* < 0.01); let-7b-3p expression was generally significant (*P* < 0.05), whereas miR-508-3p and miR-891a-5p expression differences were not statistically significant (*P* > 0.05). Among them, the miR-193b-3p expression differences were inconsistent with the direction of sequencing results, although they were significant. Therefore, miR-125b-2-3p, miR-1262, let-7b-3p, miR-548o-3p, and miR-205-5p were finally selected for the next step of target gene prediction and KEGG pathway and GO enrichment analyses. The relative expression levels of the five differentially expressed miRNAs are shown in Figure 3C.

### KEGG and GO analyses of differentially expressed miRNAs

Based on RNA sequencing results and subsequent validation, we revealed that five miRNAs (miR-125b-2-3p, miR-1262, let-7b-3p, miR-548o-3p, and miR-205-5p) were differentially expressed between the two groups. The target genes of the five miRNAs were predicted using TargetScan, miRDB, and miRWalk databases. The main biological processes, molecular functions, and cellular components of the target genes of the five miRNAs were identified by GO analysis. A total of 246 meaningful GO functions were obtained, including 47 molecular functions (mainly ubiquitin-protein ligase, protein serine/threonine kinase, DNA-binding transcription factor, and protein kinase activities), 44 cellular components (mainly nucleoplasm, cytoplasm, and post-emphasis density), and 155 biological processes (mainly positive and negative regulation of RNA polymerase II promoter transcription, positive and negative regulation of gene expression, cell migration). The top 30 GO functions identified by GO annotation are shown in Figure 3B. To better understand the biological functions of the predicted target genes, KEGG analysis was performed to reveal the main pathways in which the candidate target genes might be involved. The top 20 significantly enriched pathways were identified, including the PI3K/Akt, MAPK, Rap1, and mTOR signaling pathways (Figure 3A), and classified into environmental information processing (six), molecular processes (three), organismal systems (four), and human diseases (seven).

### Correlation between clinical characteristics and urinary exosome miRNA in MsPGN patients

**Figure 4:**
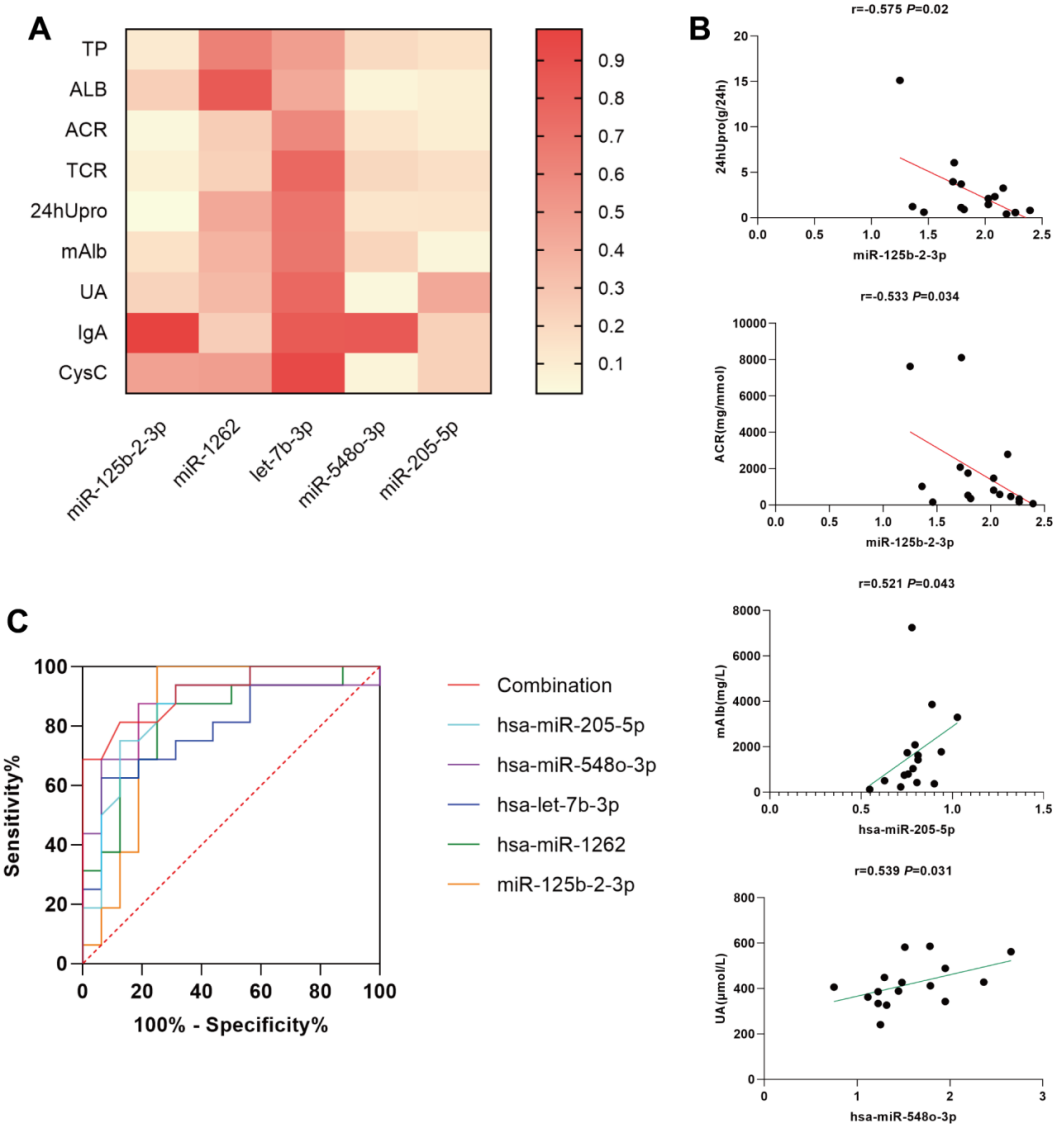
(**A**) simple linear regression between differentially expressed miRNAs and several clinical indicators in MsPGN patients. (**B**) Scatter diagram and linear correlation analysis. (**C**) Receiver operating characteristic (ROC) analysis of the combination of urinary exosomal miR-125b-2-3p, miR-1262, let-7b-3p, miR-548o-3p, and miR-205-5p for discriminating MsPGN. Plasma miRNAs yield an area under the ROC curve of 0.916.

**Figure 5:**
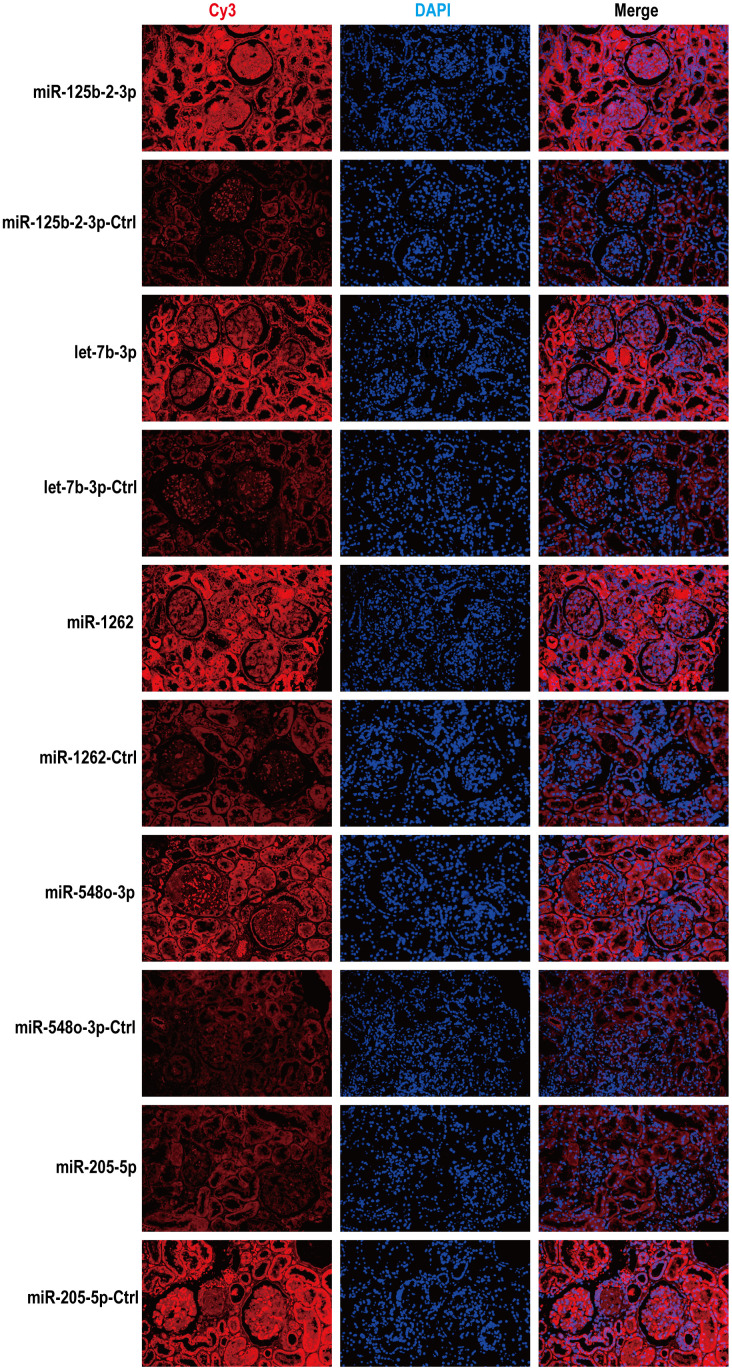
**According to fluorescence *in situ* hybridization, all five miRNAs were expressed in renal tissues of MsPGN patients.** Nuclei are stained blue (DAPI) and miRNAs are stained red. Magnification, x200.

Spearman order correlation analysis was used to analyze the correlations between the five exosomal differential miRNAs (miR-125b-2-3p, miR-1262, let-7b-3p, miR-548o-3p, and miR-205-5p) and several clinical indicators in MsPGN patients. A *P*-value < 0.05 indicated correlation, and the larger the *r*-value, the greater the correlation. Both ACR and 24hUpro were inversely correlated with miR-125b-2-3p expression. Additionally, there was a positive correlation between uric acid levels and miR-548o-3p. miR-205-5p was positively correlated with urinary microalbumin. TP, ALB, TCR, IgA, and CysC were not correlated with any of the differentially expressed miRNAs (Table 3 and Figure 4). Meanwhile, fluorescence *in situ* hybridization was performed to detect miRNAs expression in renal tissues of MsPGN patients. The expression of miR-205-5p, let-7b-3p, miR-1262, and miR-548o-3p was elevated in the MsPGN patient group, and miR-125b-2-3p was decreased in the MsPGN patient group (Figure 5).

**Table 3:** Summary of statistically significant linear correlations between miRNAs in patients with MsPGN and laboratory measurements.

	**miR-125b-2-3p**		**miR-1262**		**let-7b-3p**		**miR-548o-3p**		**miR-205-5p**	
	***P***	***r***	***P***	***r***	***P***	***r***	***P***	***r***	***P***	***r***
TP (g/dL)	0.106	0.42	0.633	-0.13	0.478	-1.91	0.192	-0.343	0.154	-0.374
ALB (g/dL)	0.244	0.309	0.841	0.054	0.424	-0.215	0.052	-0.494	0.083	-0.046
ACR (mg/mmol)	0.034	-0.533	0.259	-0.3	0.587	0.147	0.14	0.386	0.092	0.435
TCR (mg/mmol)	0.08	-0.45	0.24	-0.312	0.762	0.082	0.201	0.337	0.169	0.362
24hUpro (g/24 h)	0.02	-0.575	0.431	-0.212	0.696	0.106	0.138	0.387	0.144	0.382
mAlb (mg/L)	0.154	-0.374	0.38	-0.235	0.688	0.109	0.221	0.324	0.043	0.521
UA (μmol/L)	0.23	-0.318	0.35	-0.25	0.762	-0.082	0.031	0.539	0.431	0.212
IgA (g/L)	0.981	0.007	0.253	-0.304	0.837	-0.056	0.847	-0.052	0.241	-0.311
CysC (mg/L)	0.467	-1.96	0.478	0.191	0.935	0.022	0.056	0.487	0.239	0.312

## DISCUSSION

The current study identified the urinary exosomal differential miRNA profile of MsPGN patients. The results revealed statistically significantly increased miR-125b-2-3p, miR-1262, let-7b-3p, and miR-548o-3p and decreased miR-205-5p. Differential miRNAs target genes regulate genes involved in cancer and PI3K/Akt, MAPK, Rap1, and mTOR signaling pathways. The expression levels of the five miRNAs were mainly correlated with 24-h urine protein quantification and ACR, mAlb, and UA levels.

Among these differential miRNAs, miR-205-5p has been previously associated with renal diseases and is involved in angiogenesis and regulates related cellular signaling pathways such as cell migration, proliferation, and apoptosis [16]. Jie et al. found that circ-ACTR2 levels were upregulated in diabetic kidney disease and human renal mesangial cells (HRMCs) treated with high glucose. Silencing circ-ACTR2 expression partially abrogated high-glucose-induced cell proliferation, inflammation, ECM accumulation, and oxidative stress in HRMCs [17]. In addition, miR-205-5p directly targets the high mobility group AT-hook2 (HMGA2), and HMGA2 downregulation protects against high-glucose-treated HRMCs injury. Circ-ACTR2 mediates high-glucose-induced HRMC injury through the miR-205-5p/HMGA2 axis. In addition, miR-205-5p is reportedly involved in renal clear cell carcinoma and colorectal cancer cell proliferation, invasion, and migration [18, 19]. In the present study, urinary exosomal miR-205-5p was downregulated in MsPGN patients, consistent with its expression trend in diabetic kidney disease. Furthermore, we found that miR-205-5p is associated with urinary microalbumin; therefore, whether miR-205-5p mediates downstream signaling pathways and thus affects thylakoid region proliferation deserves further investigation.

miR-125b, miR-1262, let-7b-3p, and miR-548o-3p were significantly upregulated. miR-125b-2-3p, miR-1262, and let-7b-3p have been primarily studied in cancer and are involved in cancer development through their target genes and downstream signaling pathways. However, miR-548o-3p has not been studied. Zeng et al. found that miR-125b-2-3p expression was significantly reduced in colorectal cancer tissues and cell lines. The high miR-125b-2-3p expression was associated with relatively lower proliferation rates and less metastasis, and functional experiments showed that miR-125b-2-3p overexpression attenuated tumor cell proliferation and epithelial-mesenchymal transition, and downregulation of its expression was associated with lower proliferation and metastasis. Moreover, miR-125b-2-3p expression promotes cell development and metastasis *in vitro* and *in vivo* [20]. In addition, Meng et al. proposed that miR-125b-2-3p expression was elevated in renal clear cell carcinoma, accelerated the migration of renal clear cell carcinoma cells, and promoted tumor metastasis by downregulating its target gene EGR1. The role of miR-125b-2-3p in different cancers is related to disease type, pathogenesis, and disease progression is a complex process regulated by multiple targets and pathways [21]. Ling et al. found that LRP8 is highly expressed in breast cancer tissues and cell lines compared with that in normal human breast tissues. The poor prognosis of breast cancer patients has been associated with the upregulation of LRP8 and negatively correlated with miR-1262 overexpression. Functionally, deletion of LRP8 in breast cancer cells impairs cell proliferation, clone formation, invasion, and migration abilities, consistent with an upregulated miR-1262 effect. Bioinformatics predictions and luciferase reporter analysis confirmed that miR-1262 is an LRP8 upstream factor and negatively regulates LRP8 expression. Therefore, miR-1262-regulated LRP8 can regulate cell proliferation and migration and other processes to promote breast cancer development [22]. Sun et al. reported that has-miR-1262 expression was significantly increased in exosomes from septic patients and regulated apoptosis and glycolysis in human cardiomyocytes via the has-miR-1262/SLC2A1 signaling pathway [23]. Li et al. found that Let-7b-3p is downregulated in lung adenocarcinoma cells and tissue samples, and low Let-7b-3p expression is associated with poor prognosis in lung adenocarcinoma patients. Furthermore, Let-7b-3p inhibits proliferation and metastasis of lung adenocarcinoma cells *in vivo* and *in vitro* by directly targeting the BRF2-mediated MAPK/ERK pathway [24]. In summary, current studies on miR-125b-2-3p, miR-1262, and let-7b-3p are mainly involved in cancer development by interfering with downstream signaling pathways to regulate biological processes such as cell proliferation and migration and apoptosis. In the present study, miR-125b-2-3p, miR-1262, and let-7b-3p were significantly highly expressed in urinary exosomes of MsPGN patients. Whether these miRNAs mediate pathological MsPGN damage by interfering with downstream-related signaling pathways to regulate cell proliferation and migration, apoptosis, and other biological processes warrants further study.

To verify the function of these differential miRNAs in MsPGN patients, we performed GO and KEGG analyses. The biological processes primarily involved the negative regulation of RNA polymerase II promoter transcription, positive regulation of DNA template transcription, and cell migration. Moreover, several enriched pathways were indicated, among which PI3K/Akt, MAPK, Rap1, and mTOR signaling pathways were listed as the top 4. The proliferation of GMCs in MsPGN appears to be critical for the subsequent increase in the ECM and glomerulosclerosis development [25, 26]. Activation of the PI3K/Akt signaling pathway has been shown to be associated with cell proliferation and ECM synthesis [27–29]. Regarding PI3K/Akt signaling pathway, numerous studies have demonstrated its important regulatory role in GMCs proliferation, and Liu et al. showed that paeoniflorin could effectively reduce 24-h urinary protein in MsPGN rats. The protective effect of paeoniflorin was accompanied by strong inhibition of the PI3K/AKT/GSK-3β pathway. Paeoniflorin enhanced the inhibitory effect of the PI3K inhibitor LY294002 and inhibited the activation of the PI3K/AKT/GSK-3β pathway by the PI3K agonist insulin-like growth factor 1 (IGF-1), thus downregulating the PI3K/AKT/GSK-3β pathway to inhibit thylakoid cell proliferation and the inflammatory response and ameliorate pathological injury in MsPGN [30]. Li et al. found that PAQR3 expression is significantly upregulated in human thylakoid cells under a high glucose environment [31]. PAQR3 knockdown effectively inhibits proliferation and ECM production and significantly reverses PI3K/AKT pathway activation of HRMCs stimulated by high glucose. The important role of MAPK in mediating apoptosis by activating apoptosis-related genes is supported by extensive evidence [32, 33]. Sublytic C5b-9 induces apoptosis via MEKK2-p38 MAPK-IRF-1-TRADD-Caspase 8 in rat Thy-1 nephritis in GMCs [34]. The inflammatory response is thought to be an important factor in the MsPGN development [35, 36]. C5α is a potent pro-inflammatory mediator that correlates with the severity of various renal diseases and induces the synthesis of IL-6 and TNF-α in rat GMCs through MAPK signaling pathway activation [37]. Rap1 (also known as Krev-1) is a small GTPase belonging to the Ras superfamily that is involved in the formation and stabilization of E-cadherin-based cell–cell adhesion in epithelial cells [38–40]. Thus, Rap1 is a key mediator of integrin-mediated cell–ECM adhesion [41].

In addition, we performed a clinical analysis using Spearman correlation and simple linear regression analyses to elucidate the relationship between each differentially expressed miRNA and various MsPGN clinical features. According to our findings, both ACR and 24hUpro showed significant negative correlations with miR-125b-2-3p. Moreover, there was a positive correlation between uric acid levels and miR-548o-3p, and miR-205-5p was positively correlated with urinary microalbumin. Since miRNA expression correlated with different clinical features of MsPGN, we combined these five miRNAs in ROC analysis with an area under the curve of 0.916, suggesting high diagnostic efficacy.

## CONCLUSIONS

In this study, we measured the urinary exosomal miRNA profile of MsPGN. Exosomal miRNAs can be fully carried and released into target cells to regulate subsequent transcriptional processes. This cell–cell-mediated and epigenetic regulation could better explain the phenotype of MsPGN rather than genetic variation. Moreover, this study is the first report on the differential expression profile of urinary exosomal miRNAs in MsPGN patients. Considering miRNA-driven disease regulation, future translational research in this field may provide new diagnostic and therapeutic approaches to treat MsPGN. However, some limitations remain. The cohort in the present study is relatively small; thus, the results need to be validated in a larger population with other specific phenotypes. Nevertheless, the present study extends our understanding of the urinary exosomal miRNA expression profile in MsPGN patients, and the aberrant epigenetic regulation increases the risk of the syndrome and may lead to complications, with specific mechanisms and target cells and tissues to be elucidated by further studies.

## MATERIALS AND METHODS

### Study participants

All MsPGN patients were from the wards and outpatient clinics of the Department of Nephrology, the First Affiliated Hospital of Anhui University of Chinese Medicine (October 2019–October 2021), and baseline demographic and clinical data were recorded at the time of renal biopsy. The control group was derived from sex- and age-matched healthy subjects (from healthy volunteers at the medical screening center). Patients were all aged 18–70 years; five were MsPGN patients, and five were controls enrolled as the discovery cohort for exosomal miRNA sequencing. An additional 16 participants were enrolled in each group, increasing the validation cohort to 21 MsPGN patients vs. 21 controls. Data for all participants were obtained with their informed consent, and the study was approved by the ethical review committee of the Anhui University of Chinese Medicine.

### Exosome isolation and RNA extraction

The morning urine of patients with MsPGN and that of normal healthy subjects was collected in 50-mL centrifuge tubes and sent to the laboratory within 1 h for preparation for centrifugation. Subsequently, the urine samples were centrifuged at 500 × *g* at 4° C for 10 min, after which the sediment was removed and the remaining supernatant transferred to new 50-mL sterile centrifuge tubes. The supernatant from the previous step was then centrifuged at 2,500 × *g* at 4° C for 30 min. After centrifugation, cells and debris were removed, the sediment discarded, and the supernatant transferred to new 50-mL Beckman special centrifuge tubes (Beckman Coulter, Brea, CA, USA). These were then centrifuged at 17,000 × *g* at 4° C for 30 min to remove large vesicles, after which the sediment was discarded. The resulting supernatant was transferred to new Beckman centrifuge tubes and centrifuged at 110,000 × *g* for 80 min at 4° C; the granular precipitate obtained at the bottom of the tube was the exosome. After resuspending the precipitate from the previous step with PBS, it was centrifuged at 110,000 × *g* for 80 min at 4° C, and the pellet-like precipitate at the bottom of the tube was the purified exosome. This was carefully blown with 100 μL of PBS for resuspension, after which the exosome suspension was aspirated into an EP tube and stored at -80° C in a refrigerator.

Total RNA was extracted using a mirVana miRNA isolation kit (Thermo Fisher Scientific, Waltham, MA, USA) according to the manufacturer’s protocol and quantified using a Nanodrop2000 Spectrophotometer (Thermo Fisher Scientific). RNA integrity was assessed using an Agilent 2100 Bioanalyzer (Agilent Technologies, Santa Clara, CA, USA).

### MicroRNA library construction and sequencing

One microgram of total RNA per sample was used for small RNA library construction using a TruSeq small RNA sample preparation kit (Cat. No. RS-200-0012; Illumina, San Diego, CA, USA). Briefly, total RNA was ligated to the adapters at both ends, after which the splice ligated RNA was reverse transcribed to cDNA and PCR amplified. Thereafter, the small RNA libraries were isolated and purified from the 140–160-bp PCR products. Library quality was assessed using an Agilent Bioanalyzer 2100 system (Agilent Technologies) with a DNA high sensitivity chip. The libraries were finally sequenced using an Illumina HiSeq X Ten platform (Illumina), and 150-bp paired-end reads were generated. The sequencing of small RNA library construction and data analysis were partially conducted by OE Biotech Co., Ltd. (Shanghai, China).

### Western blot

Gel configuration was conducted according to the Takara catalog (Takara Bio Inc., Shiga, Japan). The sample stock solution was diluted to the desired loading volume, and then 5× SDS-PAGE protein loading buffer was added to a sample dilution of 1:4. Next, the proteins were fully denatured in a boiling water bath for 15 min. After cooling to room temperature, an appropriate amount (approximately 100 μg) was subjected to SDS-PAGE electrophoresis and transferred to a polyvinylidene difluoride (PVDF) membrane. Subsequently, the membrane was blocked with 5% skimmed milk. Thereafter, primary antibodies were added and diluted with primary antibody diluent at the appropriate ratios [CD9 (#Ab92726, 1:500; Abcam), CD63 (#Ab216130, 1:500; Abcam), and TSG101 (#Ab125011, 1:3,000; Abcam)] and incubated overnight at 4° C with slow shaking. Secondary antibodies included HRP-labeled Goat Anti-Mouse IgG(H+L) antibody (A0216, 1:1,000; Beyotime) and HRP-labeled Goat Anti-Rabbit IgG(H+L) antibody (A0208, 1:1,000; Beyotime). Finally, the PVDF membrane was immersed in the developing solution to develop the exposure, and the images were acquired using a UVP gel imaging analyzer (US, GelDoc-It Ts3 Imaging System).

### Quantitative real-time PCR

Total RNA was extracted from urinary exosomes using TRIzol reagent (Life Technologies, USA) and reverse transcribed to cDNA using a PrimeScript^™^ RT Reagent Kit with a gDNA Eraser (RR047A, AL21115A; Takara Bio Inc.) for miRNA expression verification. Glyceraldehyde-3-phosphate dehydrogenase (GAPDH) was used as the internal reference. All the primer sequences were designed and synthesized by Sangon Biotech Company (Shanghai, China) and data were analyzed according to the 2^-ΔΔCt^ method. Primer design as shown in Table 4.

**Table 4:** Primer sequences.

**Gene**	**Forward primer** **(5'→3')**	**Reverse primer** **(5'→3')**
U6	CTCGCTTCGGCAGCACA	AACGCTTCACGAATTTGCGT
hsa-let-7b-3p	ACACTCCAGCTGGGCTATACAACCTACTGC	TGGTGTCGTGGAGTCG
hsa-miR-125b-2-3p	ACACTCCAGCTGGGTCACAAGTCAGGCTCT	TGGTGTCGTGGAGTCG
hsa-miR-1262	ACACTCCAGCTGGGATGGGTGAATTTGTAG	TGGTGTCGTGGAGTCG
hsa-miR-205-5p	ACACTCCAGCTGGGTCCTTCATTCCACCGG	TGGTGTCGTGGAGTCG
hsa-miR-548o-3p	ACACTCCAGCTGGGCCAAAACTGCAGTTAC	TGGTGTCGTGGAGTCG

### Transmission electron microscopy

The exosome samples were removed from the -80-° C refrigerator, placed in an ice box, dissolved, and centrifuged, after which 15 μL were pipetted onto a copper grid for 1 min. Next, the exosome samples were blotted dry on the copper grid using filter paper, and then 15 μL of 2% UO2 acetate staining solution were pipetted onto the copper grid for 1 min at room temperature. Finally, the finished samples were baked under a lamp for 10 min, and the final images were observed using a transmission electron microscope at 80 kV (Tecnai, G2 spirit; FEI Company, Hillsboro, OR, USA), photographed, and saved.

### Nanoparticle tracking analysis

The sample cells were washed with ultrapure water, and the instrument (NanoFCM, N30E) was calibrated using polystyrene microspheres (100 nm). Next, the exosomes were removed and diluted to the appropriate magnification. The instrument should be tested with the standard before the sample is loaded, and the sample should be diluted in a gradient to avoid blocking the injection needle. The particle size and concentration information of exosomes can then be obtained after the sample is tested.

### Fluorescence *in situ* hybridization

The expression levels and localization of 5 miRNAs in renal tissues were detected by fluorescence *in situ* hybridization (FISH). Five probes for Cy3-labeled miRNAs were designed (hsa-miR-125b-2-3p, 5'-GTCCCAAGAGCC+TGACT+TGTGA-3'; hsa-miR-1262, 5'-ATCCT+ TCTACAAAT+TCACCCAT-3'; hsa-let-7b-3p, 5'-GGGAAGGCAG+TAGGT+TGTATAG-3'; hsa-miR-548o-3p, 5'-GCAAAAGTAAC+TGCAGT+TTTGG-3'; hsa-miR-205-5p, 5'-CAGAC+TCCGG+TGGAATGAAGGA-3', purchased from GenePharma (Shanghai, China). Each probe was hybridized overnight according to the manufacturer's instructions.

### Bioinformatics analysis

To predict the target genes of differential miRNAs and take their intersection as candidate target genes, TargetScan Human version 7.2 (http://www.targetscan.org/vert_72/), MicroRNA Target Prediction Database (miRDB; http://mirdb.org/), and miRWalk (http://mirwalk.umm.uni-heidelberg.de/) were used. Gene ontology (GO; http://www.geneontology.org) analysis was then performed to analyze the main functions of the putative target genes, and Kyoto Encyclopedia of Genes and Genomes (KEGG; https://www.genome.jp/kegg) analysis was used to identify potentially altered molecular pathways.

### Statistical analyses

Statistical analyses were performed using SPSS (version 25.0; SPSS Inc., Chicago, IL, USA). Continuous variables were tested for abnormality using the Kolmogorov–Smirnov test. To determine statistical significance, normally distributed variables were analyzed using Student’s *t*-test, and nonparametric data were assessed using the Wilcoxon–Mann–Whitney test. Receiver operating characteristic (ROC) curve analysis was used to assess the diagnostic potential of plasma exosomal miRNAs to differentiate MsPGN samples from controls. Logistic regression analysis was used to assess the diagnostic efficacy of miRNA combinations. Spearman correlation analysis was performed to assess the correlation between miRNA expression and MsPGN phenotype. *P* < 0.05 was considered statistically significant.
